# A turn-on fluorescent solid-sensor for Hg(II) detection

**DOI:** 10.1186/1556-276X-9-431

**Published:** 2014-08-26

**Authors:** Mayela De la Cruz-Guzman, Angelica Aguilar-Aguilar, Luis Hernandez-Adame, Alan Bañuelos-Frias, Francisco J Medellín-Rodríguez, Gabriela Palestino

**Affiliations:** 1 Biopolymers and Nanostructures Laboratory, Faculty of Chemical Sciences, Universidad Autónoma de San Luis Potosí, Av. Manuel Nava No. 6, San Luis Potosí, San Luis Potosí 78210, México; 2 Materials Laboratory, Faculty of Chemical Sciences, Universidad Autónoma de San Luis Potosí, Av. Manuel Nava No. 6, San Luis Potosí, San Luis Potosí 78210, México; 3 Colloids and Interfaces Laboratory, Institute of Physics, Universidad Autónoma de San Luis Potosí, Av. Manuel Nava No. 6, San Luis Potosí, San Luis Potosí 78210, México

**Keywords:** Chemosensor, Porous silicon, Rhodamine derivative, Fluorescence, Heavy metal

## Abstract

A rhodamine organosilane derivative (Rh-UTES) has been obtained by one-pot synthesis. The chemical structure of Rh-UTES was confirmed by nuclear magnetic resonance (NMR) and infrared (FTIR) techniques. To obtain an inorganic-organic hybrid sensor, Rh-UTES was covalently immobilized on a porous silicon microcavity (PSiMc) via triethoxysilane groups. The attachment of the organic derivative into PSiMc was confirmed by FTIR, specular reflectance, and scanning electron microscopy (SEM). The optical performance of Rh-UTES receptor for Hg^2+^ detection was investigated by fluorescent spectroscopy and microscopy. Upon the addition of increasing amounts of Hg^2+^ ions, a remarkable enhancement in emission intensity was produced in both systems. In the solid phase, an increase of integrated fluorescent emission of 0.12- and 0.15-fold after Hg^2+^ receptor coordination was observed. The light harvesting capability of PSiMc devices allowed obtaining an enhanced fluorescent emission after Rh-UTES immobilization (277-fold). The fluorescence microscopy of hybrid PSiMc sensor provided an optical qualitative test for Hg^2+^ detection.

## Background

The toxicity of mercury (Hg) and its complex forms on ecosystems and human health is well known. The need to create new sensitive and practical analytical methods to detect the mercury ions in different sources has increased. Recently, ion-selective sensors have attracted attention due to their diverse potential applications as tools for the quantitative and qualitative monitoring of metal ions in many biological and environmental processes [1-6]. Ion-selective sensors could find applicability in monitoring metal ion concentrations and can be practical solutions to monitor industrial waste effluent streams and potable water. Emphasis has been placed on compound development that selectively responds to the presence of specific metal ions through a change in one or more properties of the system, such as redox potentials [7], absorption [8], or fluorescence spectra [9]. Such sensors based on ion-induced changes in fluorescence appear to be particularly attractive due to their simplicity, high sensitivity, high selectivity, and instantaneous response [10]. Fluorescent chemosensors based on xanthenes and related derivatives for the Hg^2+^ ions detection have been increasing due to the low cost and high applicability in industrial and biological processes [11]. During recent years, novel inorganic-rhodamine hybrid sensors have been published. The rhodamine derivatives have been immobilized into the different inorganic receptors. Huang et al. reported fluorescent gold nanoparticle sensors for detection of Hg^2+^ ions [12]. Since gold nanoparticles (AuNPs) are highly efficient fluorescence quenchers, the rhodamine derivative had to be released from the AuNPs to restore the rhodamine fluorescence. Lee et al. and Zhou's group developed a covalently bonded mesoporous silica rhodamine derivative [13,14]. Childress and co-workers reported dye-doped polymer nanoparticles that are able to detect mercury ions. The nanoparticles were prepared by precipitation of highly fluorescent conjugated polymers and doped with rhodamine derivatives [15]. Recently, Wang and Gao designed a mercury sensor using β-NaYF_4_:Yb^3+^/Eu^3+^ nanorods as the excitation source and a rhodamine derivative as a probe [16]. In this proposal, our research group has designed a new functional rhodamine derivative (Rh-UTES) that acts as a receptor of heavy metal ions. The Rh-UTES derivative was covalently bonded to porous silicon microcavity (PSiMc) to develop a hybrid sensor. The main advantage of the proposed method is the simplicity of the system and the fact that the hybrid sensor should be easy to carry for field applications. The PSiMc has proven to be a suitable material with unique optical properties for the development of this kind of fluorescent sensor [17]. Our previous approaches in this field have shown that the detection of fluorescent molecules is possible using the optical properties of specific PSi structure (mirror or microcavity) [18]. Increased excitation and enhanced emission, both driven by the efficient reflection of light and resonance effects within the PSi microcavities, allowed the enhancement of the fluorescent response of the Rh-UTES derivative even at low molecular concentration. Hence, the variation of this method was used here to produce detection of low concentrations of heavy metals by forming metallic complexes within the pores that turn on the luminescence emission.

## Methods

Rhodamine base, ethylenediamine, *m*-xylenediisocyanate, 3-aminopropyltriethoxysilane (APTES), hydrochloric acid, hydrofluoric acid, nitric acid, sodium hydroxide, and mercury nitrate were purchased from Sigma-Aldrich (St. Louis, MO, USA). All solvents were analytical reagent grade and used as received.

### Instruments and spectroscopy measurements

The reflectivity spectra were recorded in an Agilent Cary 60 UV-Vis spectrophotometer (Agilent Technologies, Sta. Clara, CA, USA) coupled with a 30° specular reflection unit. PSi samples were illuminated with the xenon source, and the reflected beam was detected with the silicon diode detector. The resulting spectra were captured in the range from 500 to 900 nm. The fluorescence images of PSiMc/Rh-UTES sensor were recorded in a Nikon Optiphot-2 fluorescence microscope (super high pressure mercury lamp power supply; Nikon, Tokyo, Japan). The Fourier transform infrared spectra (FTIR) were recorded in a Bruker Tensor 27 spectrophotometer (Bruker Corporation, Billerica, MA, USA), with 128 scans and 4-cm^-1^ resolution, coupled with a diamond crystal attenuated total reflectance unit (ATR). Nuclear magnetic resonance (NMR) measurements of ^1^H and ^13^C were carried out in a Bruker 500 MHz spectrometer. Scanning electron microscopy (SEM) was performed using a UHR dual-beam FEI Helios Nanolab 600 field emission scanning electron microscope (FEI Company, Hillsboro, OR, USA). Samples were mounted on a conductive carbon tape. Images were captured at magnifications of × 20,000 and × 25,000.

### Synthesis of porous silicon

PSi samples were prepared by the wet electrochemical etching process using high-doped p-type (boron-doped) silicon wafers (thickness 500 to 550 μm) with 0.001 to 0.005 Ω cm resistivity, and with the crystallographic orientation of (100), purchased from WRS Materials (San Jose, CA, USA). The electrolyte consisted of hydrofluoric acid (48 wt%) and ethanol in the volumetric ratio of 3:7. The anodization time and current density were controlled by a computer-interfaced electronic circuit. The samples were fabricated at room temperature, and freshly etched samples were washed with ethanol and dried with pentane. To perform this work, we have selected a PSiMc, mainly due to its optical features in the reflectance spectra that allows the detection of infiltrated material into the porous structure. PSiMc configuration consists of an active porous layer embedded between two multilayered mirrors (Bragg reflectors). The PSiMc was produced by alternating layers of high porosity (H; refractive index, *n* = 1.14395) and low porosity (L; *n* = 1.25865), with current densities of 70 and 30 mA/cm^2^. Anodization times of 6.35 and 10.67 s for H and for L, respectively, were used for the fabrication of the corresponding dielectric Bragg mirrors. The PSiMc structures were fabricated with the configuration of (HL) × 5 HH (LH) × 5, where (HL) × 5 corresponded to the first Bragg reflector, HH to the cavity and (LH) × 5 to the second Bragg reflector. The PSiMc samples were thermally oxidized at 600°C for 30 min in O_2_ atmosphere to stabilize and protect them against environmental contaminants and/or natural aging [19].

### Synthesis of rhodamine fluorescent derivative

**Figure 1:**
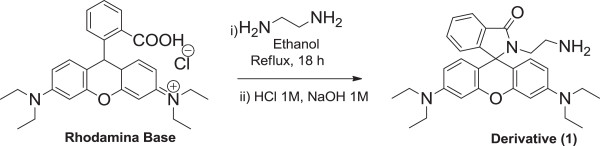
Synthesis to obtain derivative (1).

Herein, we synthesize a new rhodamine fluorescent derivative Rh-UTES bearing urea groups. To obtain this compound, several steps were needed. For the synthesis of Rh-amine derivative (1), following the procedure in the literature [20], rhodamine base (4.0 g, 8.3 mmol) and ethylendiamine (4.2 g, 70 mmol) were dissolved in EtOH (210 mL) and refluxed for 18 h. The solvent was removed by evaporation, and the residue was dissolved in an aqueous HCl solution (1 M, 333 mL). An aqueous NaOH solution (1 M) was added carefully to the solution with magnetic stirring. The precipitate was recovered by filtration, washed thoroughly with water, and then dried under vacuum, yielding (1) as a pink fluffy powder (3.21 g, 80%); ^1^H NMR (CDCl_3_): δ (ppm) 7.85 (d, 1H, *J* = 2.5 Hz), 7.44 (t, 2H, *J* = 6.7 Hz), 7.06 (s, 1H), 6.42 to 6.37 (m, 6H), 3.33 (q, 10H, *J* = 7.1 Hz), 2.91 (t, 2H, *J* = 6.7 Hz), 1.16 (t, 12H, *J* = 6.7 Hz); ^13^C NMR (CDCl_3_): δ (ppm) 170.5, 153.7, 153.3, 149.1, 133.2, 130.0, 128.4, 128.3, 123.9, 123.2, 108.6, 103.6, 97.8, 66.4, 44.4, 41.1, 39.5, 12.66. Figure 1 shows the synthesis to obtain derivative (1)*.*

**Figure 2:**
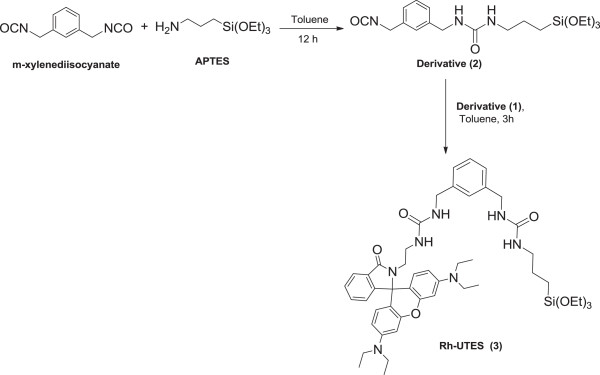
Synthesis of Rh-UTES (3).

The Rh-UTES derivative was obtained by following the next procedure (Figure 2): In a 10-mL round-bottom flask fitted with magnetic stirrer, *m*-xylenediisocyanate (0.05 g, 0.26 mmol) and 3-aminopropyltriethoxysilane (APTES) (0.04 g, 0.18 mmol) were refluxed in 5 mL of toluene under N_2_ for 12 h. Derivative (2) was used without isolation, the Rh-amine derivative (1) was added (0.1 g, 0.21 mmol) under N_2_, and the reaction was refluxed for 3 h. The solvent was evaporated under reduced pressure to give a beige powder (0.22 g, 96%); ^13^C NMR (DMSO-*d*_6_): δ (ppm) 168.0, 158.1, 154.2, 153.0, 148.1, 141.0, 133.2, 130.5, 128.6, 128.5, 126.2, 126.1, 126.0, 125.9, 125.7, 124.0, 122.8, 108.3, 105.3, 97.8, 64.6, 60.2, 44.1, 43.4, 40.6, 38.4, 21.2, 15.1, 14.5, 12.8; IR data: *ν*_max_ (cm^-1^): 3331, 2970 to 2890, 1695, 1624, 1574, 1513, 1082, 962, 771.

### PSi device functionalization

The binding of Rh-UTES derivative within the PSi nanostructured devices was performed following one-step method through silane chemistry by reacting the methoxy groups (-OCH_3_)_3_ of the fluorescent molecule with the siloxane (-Si-O) groups of the thermally oxidized PSi surface [18]. Briefly, the PSi samples were dipped in 2 mL of Rh-UTES derivative solution (1.16 μM in ACN) at room temperature, and all of the reaction system was kept under inert atmosphere with magnetic stirring. The reaction time was fixed at 3 h to obtain the final PSiMc/Rh-UTES sensors.

### Metal capture

Once obtained, the PSiMc/Rh-UTES sensors were exposed to 2.0 mL of mercury aqueous solutions. To assure the presence of the free Hg^2+^ ions, the solutions were adjusted at pH 3.0 using HNO_3_ 0.1 M (based in the Hg speciation diagram). The complexation reactions were carried out at room temperature for 12 h under magnetic stirring.

## Results and discussion

**Figure 3:**
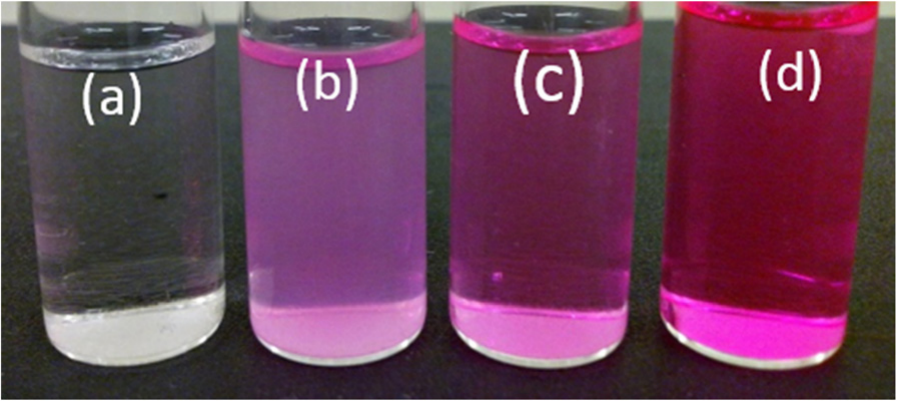
**Colorimetric changes in the Rh-UTES derivative solutions. (a)** Before Hg^2+^ addition and after Rh-UTES-Hg^2+^ complex formation at the following molar ratios: **(b)** 1:1, **(c)** 1:6, and **(d)** 1:10, respectively. Rh-UTES concentration remained fixed at 1.16 μM in ACN solution.

Rh-UTES derivative was successfully synthesized from a rhodamine base in a relatively good yield. To evaluate the metal ion binding capability of this new compound, a colorimetric evaluation was performed in a liquid phase. Figure 3 shows the optical behavior of the fluorescent chemosensor in solution (1.16 μM in ACN). It was observed that after the Hg^2+^ addition, the colorless solution immediately becomes pink. It is interesting to notice that the color intensity of the solution is linearly dependent on the metal concentration. The color change in the chemosensor solution after Hg^2+^ addition is attributed to the chelator-metal binding. Thus, the colorimetric change produced during Hg^2+^ capture can be used as ‘naked-eye’ detection of this metallic contaminant in solution.

**Figure 4:**
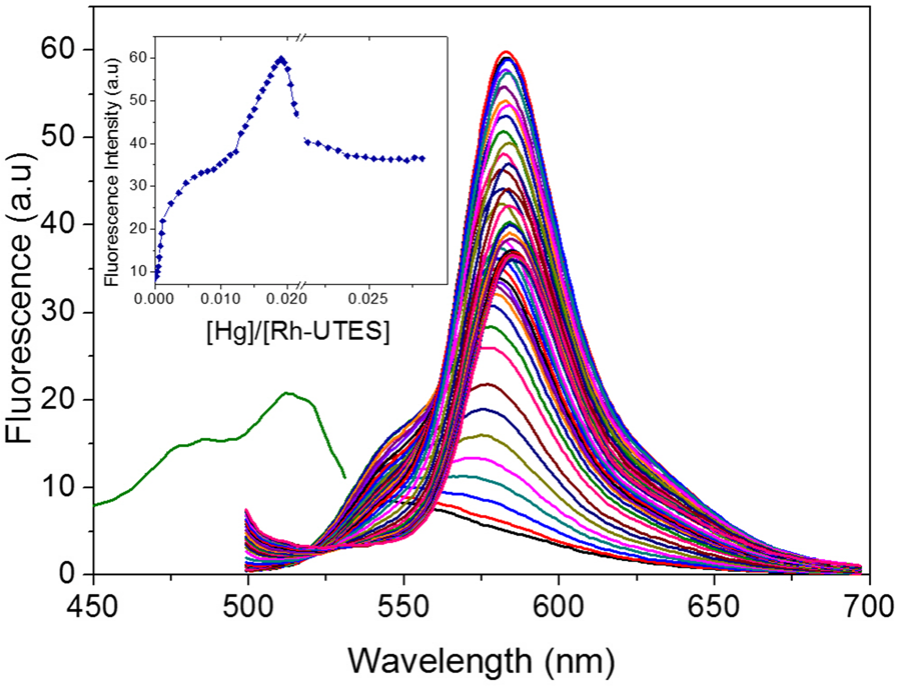
**Fluorescence response of Rh-UTES derivative in liquid phase at different metal concentration.** Fluorescence response of Rh-UTES derivative in liquid phase (1 mM in ACN) upon addition of different concentrations of Hg^2+^ ions (0.166 to 27.0 μM). *λ*_exc_ = 485 nm. The inset shows the fluorescence intensity of the Rh-UTES-Hg^2+^ complex as a function of [Hg^2+^]/[Rh-UTES] ratio.

The photoluminescent properties of Rh-UTES derivative in solution were investigated toward the metal ion complexation. Figure 4a shows the excitation and emission spectra of Rh-UTES derivative with peaks centered at 513 and 583 nm, respectively. In the figure we can notice that the organic receptor exhibited a slight fluorescence emission. Upon the addition of increasing amount of Hg^2+^ ions (0.166 to 27.0 μM) to the solution of Rh-UTES receptor, a remarkable enhancement in the emission intensity was observed. This fluorescent enhancement is attributed to the formation of the Rh-UTES-Hg^2+^ complex. Thus, it is clear that the addition of Hg^2+^ ions ‘turns-on’ the fluorescence whereby the colorless weak fluorescent derivative changed to a colored highly fluorescent complex, as was also shown in Figure 3. Additionally, we found that the Rh-UTES-Hg^2+^ complex presents a maximum emission at 11.9 μM Hg^2+^ concentration, after which a fluorescent quenching phenomenon was observed. The fluorescent intensity is reduced since some molecules of the complex act as a quencher (because the high concentration of the complex may induce a self-absorption process) which in turn decreases the number of molecules that can emit. Finally, after addition of 24.2 μM Hg^2+^ concentration, the fluorescent emission of complex remains constant, which is attributed to the depletion of Rh-UTES derivative.

**Figure 5:**
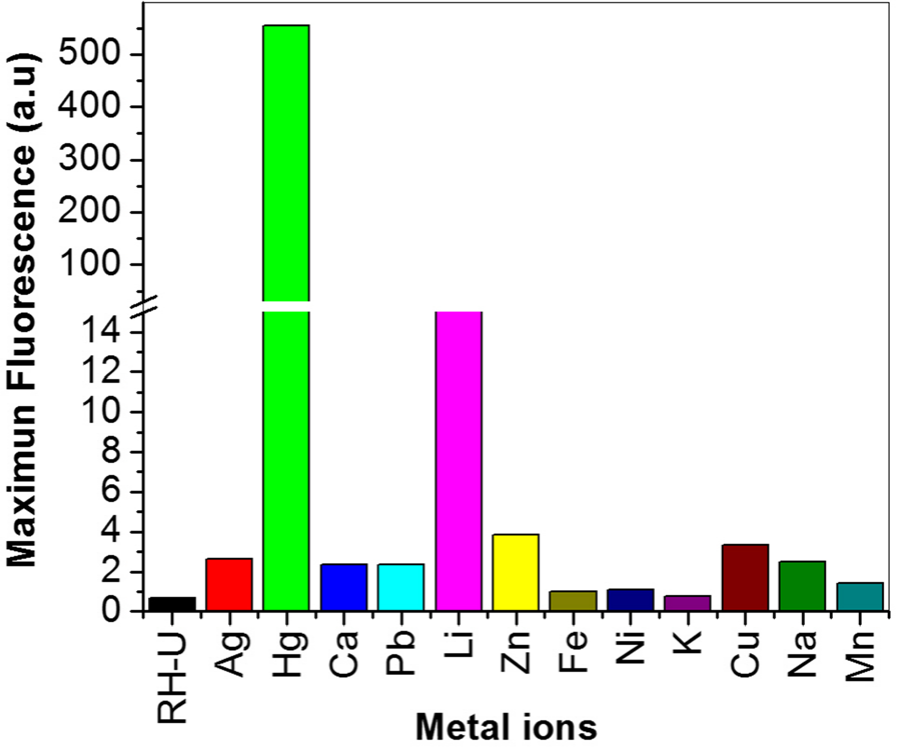
**Maximum fluorescence emission of Rh-UTES after metal capture.** Maximum fluorescence emission of Rh-UTES (10 μM in ACN) derivative upon addition of 100 μM of Ag^+^, Hg^2**+**^, Ca^2**+**^, Pb^2**+**^, Li^2**+**^, Zn^2**+**^, Fe^2**+**^, Ni^2**+**^, K^+^, Cu^2**+**^, Na^+^, and Mn^2**+**^, respectively. The emission spectra were recorded under identical experimental conditions at excitation wavelength of 485 nm.

The fluorophore selectivity was also investigated by measuring the changes in the fluorescent emission produced by the addition of the following metal ions: Ag^+^, Hg^2+^, Ca^2+^, Pb^2+^, Li^2+^, Zn^2+^, Fe^2+^, Ni^2+^, K^+^, Cu^2+^, Na^+^, and Mn^2+^ to various solutions of Rh-UTES. The results are displayed in Figure 5; it is clear that the presence of these ions led to increases in the fluorescence intensity to varying degrees. It was observed that only Li^2+^ ions promote small fluorescence intensity changes, while the other metal ions did not cause any significant changes under identical conditions. The fluorescent emission intensity observed for Hg^2+^ over the other ions is remarkably high pointing out the high selectivity of Rh-UTES toward Hg^2+^.

### Reflectance spectra

**Figure 6:**
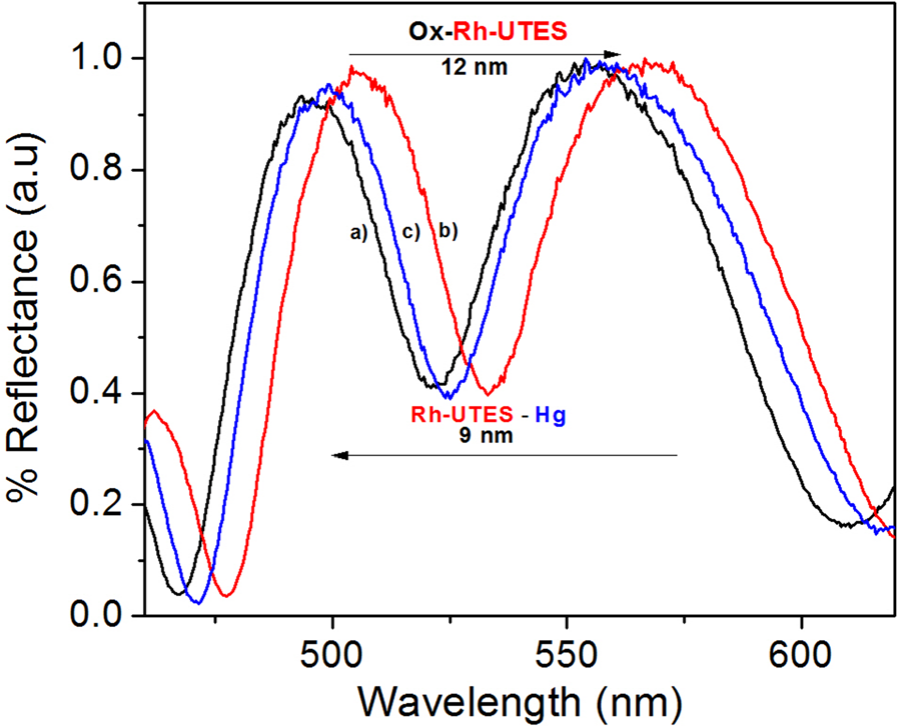
**Specular reflectance spectra of PSiMc devices.** (a) Thermally oxidized sample (black line), (b) after Rh-UTES immobilization (red line), and (c) after metal coordination (blue line). [Hg^2+^] = 3.48 μM.

The reflectance spectra of the PSiMc were recorded after each modification step using the UV-vis spectrophotometer. Figure 6 compares reflectance spectra taken before and after PSiMc functionalization and a metal capture. It is observed that Rh-UTES derivative binding produces a red shift (12 nm) in the PSiMc reflectance spectrum; we also found that this process is repeatable showing a standard deviation (SD) of ±2.12 nm. The red shift can be attributed to the effective refractive index (ȵ) changes after infiltration of the fluorescent molecule into the PSi pores [18]. After exposition of PSiMc/Rh-UTES sensor to Hg^2+^ solution, surprisingly and contrary to the expectation, a blue shift was observed in the specular reflectance spectrum (9 nm, SD ± 3.35 nm). Normally, this drift in signal (blue shifts) can be associated to the degradation (or oxidation) of PSi [21]. However, in this work, the observed negative shift is attributed to the derivative-metal binding. This was confirmed by the negative controls that were carried out to ensure the specificity of the linking chemistry. These results showed a negligible drift in the PSi sensor reflectance spectrum over the same incubation periods used to collect data in the performed experiments. It seems that the metal capture produces a decrease of ȵ. Nevertheless, to have a better understanding of the metal-ligand-substrate interactions and their effect on the optical properties of the PSiMc structure, more studies are being conducted in our research group. Thus, the capture of the metal ions for the PSi/Rh-UTES sensor was confirmed using complementary analytical techniques.

### Monitoring molecular infiltration

**Figure 7:**
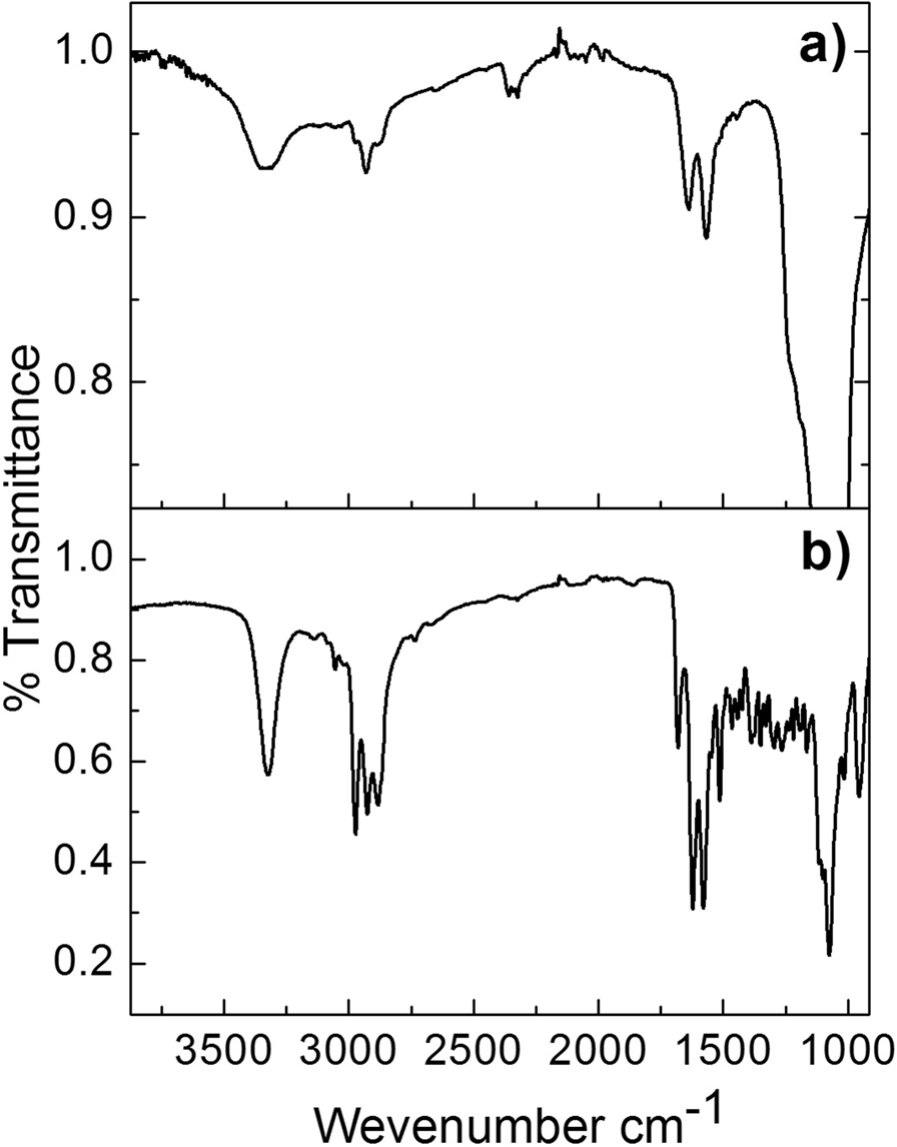
**Infrared spectra. (a)** Functionalized PSiMc/Rh-UTES device and **(b)** pure Rh-UTES derivative.

PSi nanostructured devices were analyzed by FTIR before and after derivative functionalization and the metal capture. Riikonen and co-workers reported the typical strong absorptions of oxidized PSi (OxPSi) [22]. Bands characteristic of the stretching mode of silanol groups ν(SiO-H) were observed at around 3400 cm^-1^, the δ(SiO-H) bending mode at 1640 cm^-1^, and the ν(Si-OH) stretching modes at 950 and 887 cm^-1^. An intense broad peak at around 1085 cm^-1^ was also seen, which may be due to the ν(Si-O) stretching mode for surface silicon-hydroxyl species. All of these bands are consistent with FTIR spectrum of our thermally (OxPSi) device [19]. The immobilization of Rh-UTES derivative into the PSiMc surface was carried out and confirmed by FTIR spectroscopy (Figure 7a); the hybrid sensor owns the next characteristics bands: *ν*(N-H) stretching modes at 3344 cm^-1^, *ν*(C = O) stretching modes at 2924 cm^-1^, *δ*(N-H) bending mode at 1571 cm^-1^ of secondary amide, *ν*(C-H) stretching modes of methylene groups at 3008 to 2861 cm^-1^, and mainly the siloxane (Si-O) bands of OxPSi at 1054 cm^-1^. These bands are similar to those belonging to the pure Rh-UTES derivative reported in the ‘Methods’ section (Figure 7b), thus confirming that incorporation of Rh-UTES into the PSiMc was successful. The hybrid sensor was then exposed in a Hg^2+^ solution (1.16 μM) for 12 h, and the FTIR analysis of the PSiMc/Rh-UTES-Hg^2+^ sample showed no significant changes in the infrared bands (not shown) compared with the reference spectrum of Figure 7b.

### Morphological analysis

**Figure 8:**
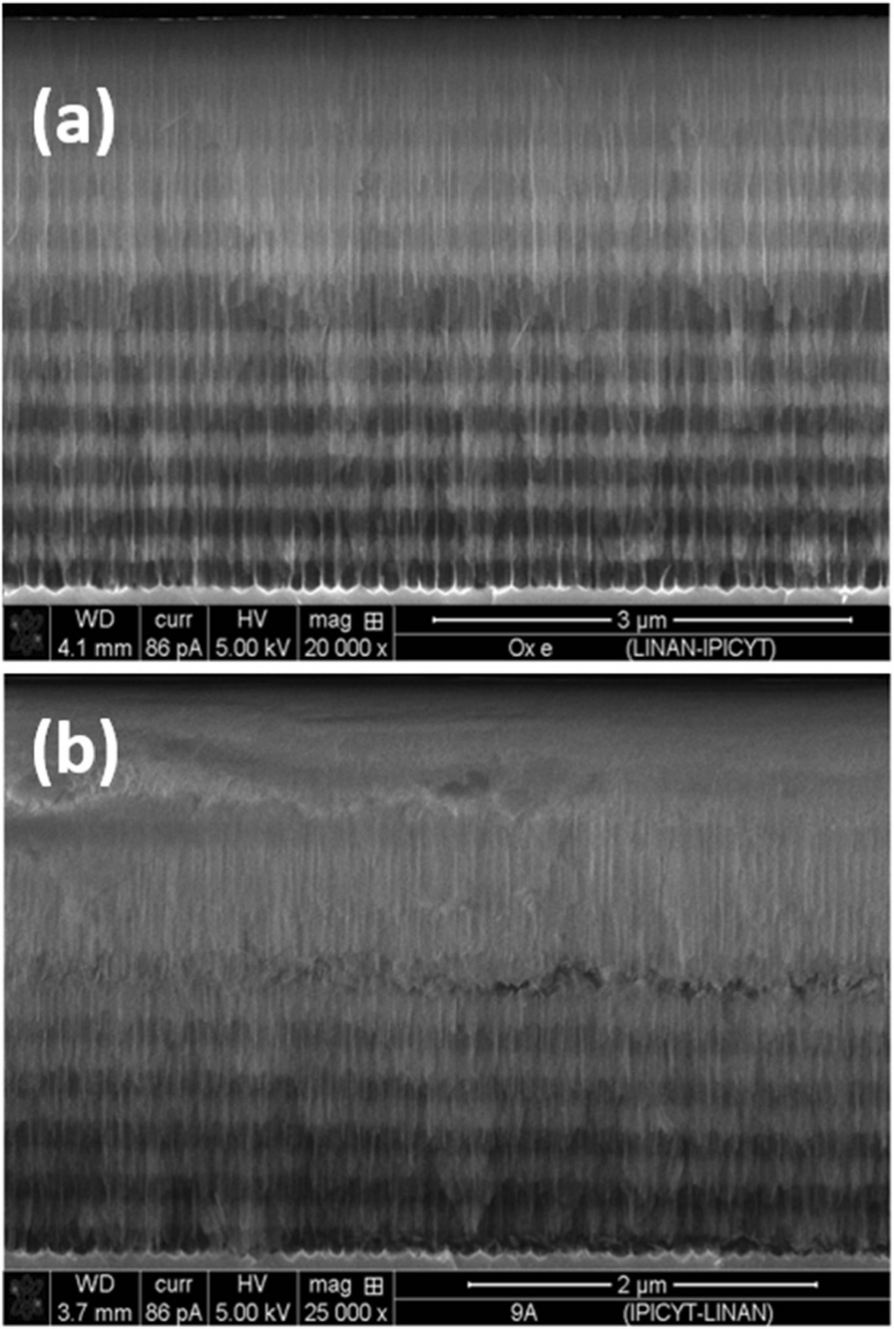
**Cross-sectional SEM micrographs of PSiMc before and after derivative immobilization. (a)** Thermally oxidized sample. **(b)** PSiMc/Rh-UTES hybrid device.

Figure 8 shows cross-sectional SEM images of PSiMc devices before (a) and after (b) functionalization with Rh-UTES derivative. The top view of unmodified PSiMc device (image not shown) shows a high porosity structure composed of well-defined pores with an average size distribution of 19.25 ± 4 nm. In these PSi structures, the pore sizes were big enough to allow the molecular infiltration as demonstrated by specular reflectance spectrometry. The lateral view of the unmodified sample (Figure 8a) shows the high (white line) and low porosity (black line) layers together with the defect layer (centered in the middle of the structure). The morphology of the PSiMc structures after chemical modification is shown in Figure 8b, and we observed a homogeneous layer of organic derivative covering the first layers of the PSi structure, which confirms the infiltration of Rh-UTES derivative into the porous device.

### Photoluminescence properties

**Figure 9:**
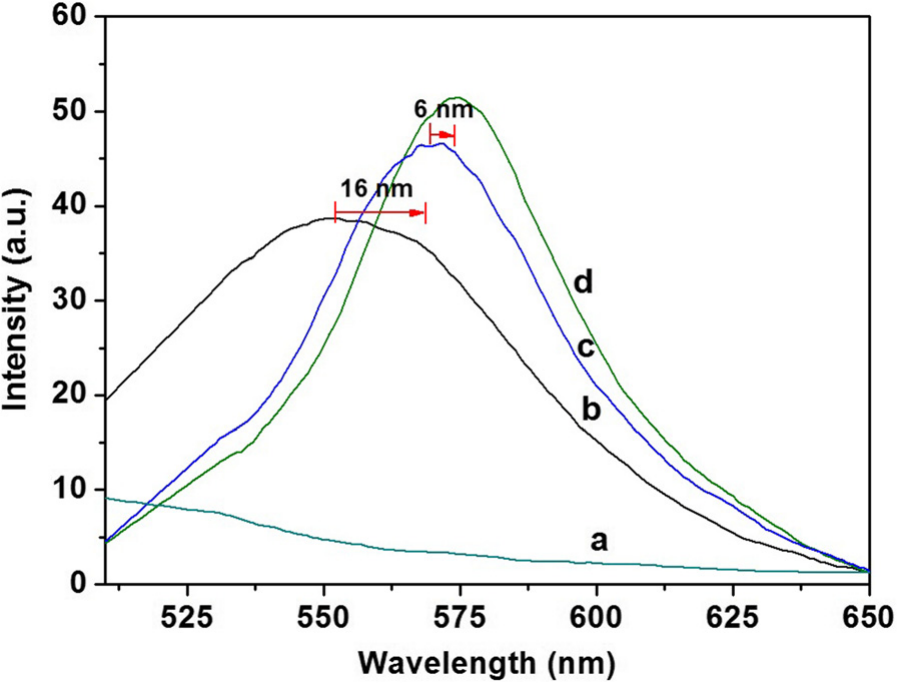
**Emission spectra of PSiMc devices (*λ***_**exc**_ **= 490 nm) before and after chemical functionalization and metal device recognition.** (a) Thermally oxidized sample, (b) PSiMc/Rh-UTES sensor (derivative (3) concentration = 1.16 μM), and (c and d) PSiMc/Rh-UTES-Hg^2**+**^ complexes (3.45 and 6.95 μM respectively).

In solid phase, photoluminescence (PL) measurements were used to characterize the performance of the fluorescent sensor under *λ*_exc_ = 490 nm. Figure 9 shows the fluorescent emission of (a) thermally oxidized PSiMc, (b) PSiMc/Rh-UTES functionalized device [1.16 μM of derivative (3)], and (c, d) PSiMc/Rh-UTES sensors after exposure to solutions contaminated with Hg^2+^ (3.45 and 6.95 μM, respectively). The amount of infiltrated derivative into the PSi pores was obtained by calculating the concentration of the residual supernatant (recovered after the exposure time of the sample was completed) and making a mass balance. The obtained concentration value was of 1.4058 ± 0.35 nmol of Rh-UTES/cm^2^ of etched area, which corresponds at approximately 20% of the initial solution concentration (1.16 μM) [19]. By comparing the optical features of bare PSiMc with that obtained after device functionalization, it is clear that the emission spectra show important optical changes. The most remarkable is the well-defined emission curve in the 525 to 625-nm range attributed to the fluorescent emission of Rh-UTES derivative, which confirms the attachment of the derivative molecule on the PSi surface. Exposure of PSiMc/Rh-UTES sensor at a heavy metal solution produced two new changes: first, an increase in the integrated emission intensity of 0.13-fold and secondly, a 16-nm red shift (552 to 568 nm) of the main peak position. As we mentioned before, some studies have demonstrated that the spirolactam-rhodamine derivatives can be used to develop liquid phase OFF-ON metal ion-fluorescent chemosensors, mainly because their chemical structure may change in the presence of metal ions. In agreement with those contributions, we believe that the enhanced emission observed when the PSiMc/Rh-UTES sensor captured the Hg^2+^ ions is produced by the formation of metal-ligand coordination bonds, which in turn induces the spirolactam ring opening [23]. Thus, based on this coordination mechanism, the red shift in the fluorescent emission may be attributed to the electronic interactions of PSiMc/Rh-UTES-Hg^2+^ complex (Figure 9c). A similar optical behavior was found in the liquid phase chemosensor; however, our solid device presents several advantages that are related with (i) the easy operation of the device, (ii) special solvents that are not needed, (iii) the higher stability of the fluorescent derivative when immobilized in the solid support, and (iv) the possibility of portability. Then, by comparing spectra (c) and (d) which correspond at the sensing of two different Hg^2+^ ion concentrations (3.45 and 6.95 μM, respectively), a 6-nm red shift (from 568 to 574 nm) and a fluorescent emission enhancement of 0.12-fold was observed. In this case, the red shift may be attributed to PSi-derivative-Hg^2+^ interaction processes produced in the reduced space of PSi pores. Our hypothesis is that after increasing the metal ion concentration, the derivative Rh-UTES receptor changed its chemical structure, provoking a molecular reorganization inside the pore. According to Tu and co-workers [24], the chemical change can reduce the distances between neighboring molecules limiting their free stretching movement and leading to their self-interaction, which may reduce their excited state energy and produce the red shift in the spectra. On other hand, the enhancement of the emission intensity observed when the PSiMc/Rh-UTES device coordinates higher amount of Hg^2+^ ions confirms that the fluorescent intensity of the PSiMc hybrid device is metal concentration dependent [25,26]. This is important to notice if the sensing principle of the PSiMc/Rh-UTES sensor is based on the fluorescence spectroscopy measurements. Finally, to investigate the optical contribution of PSi devices in the fluorescence response, we compared the fluorescence emission of Rh-UTES derivative in liquid (ACN) and immobilized on PSi structures. We observed a 277-fold fluorescence increase in the case of PSi/Rh-UTES nanostructure, and it is important to keep in mind that the derivative concentration in the solid device is three orders of magnitude lower than in the solution (1.4058 ± 0.35 nmol cm^-2^ compared with 1.16 μM). Therefore, these results highlight the benefits of use PSi optical device as support of the organic receptor.

**Figure 10:**
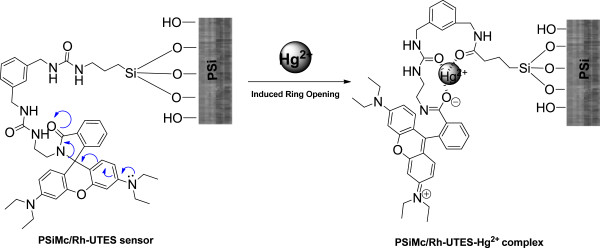
**Proposed mechanism of the coordination mode of Hg**^**2+ **^**ions.**

**Figure 11:**
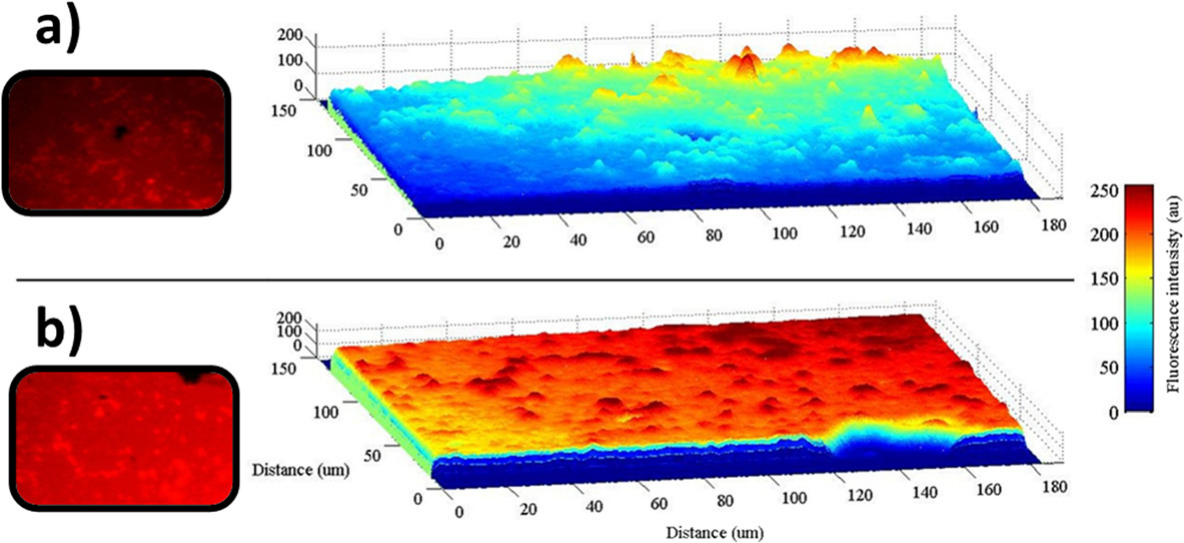
**Fluorescence emission of PSiMc sensor and its tridimensional profile before and after metal detection. (a)** PSiMc/Rh-UTES (Rh-UTES = 1.16 μM) and **(b)** PSiMc/Rh-UTES-Hg^2+^ (Hg^2+^ = 6.95 μM).

Figure 10 shows a proposed mechanism of the coordination mode of Hg^2+^ ions. Several proposed binding modes have been reported on which oxygen, sulfur, and nitrogen atoms have provided higher affinity toward Hg^2+^[11]. In our study and as the FTIR spectra have showed, two carbonyl oxygen atoms as well as the amide oxygen can provide a binding pocket for Hg^2+^. To confirm the proposed mechanism, further studies need to be completed (X-ray diffraction).An analysis using fluorescence microscopy was also carried out to characterize the emission intensity over the entire surface of the hybrid sensor. The samples were excited using a mercury lamp with 510 to 560-nm filter in a Nikon Optiphot-2 (G2-A) microscope coupled with 3CCD MTI 8-bit camera. The emission intensities are shown in the Figure 11. The image in the Figure 11a is presenting a real view of the PSiMc/Rh-UTES hybrid sensor and its corresponding tridimensional fluorescence profile over the entire surface, on which we can see the emission intensity produced for the immobilized Rh-UTES derivative. After metal sensor exposure, the hybrid sensor showed a strong brilliant red light (Figure 11b), and the fluorescence enhancement was 0.22-fold (integrated emission). This value coincided well with the fluorescent enhancement observed on the fluorescent spectroscopy analysis (0.25-fold for the same metal concentration).

## Conclusions

In this work we have proposed a novel method for detection of Hg^2+^ ions using rhodamine fluorescent derivative as the recognizing element. We studied the fluorescent performance of the derivative receptor in liquid and solid phases. In solution, after the Hg^2+^ addition to the Rh-UTES receptor, it was observed that the colorless solution becomes colored (pink) and a remarkable enhancement in the emission intensity. We found that both the color intensity and the fluorescent intensity of the solution are linearly dependent on the metal concentration. This distinct color and fluorescent change due to the spirolactam ring opening makes this derivative valuable for sensing ions through fluorescent or naked-eye detection. Additionally, a new sensing strategy was evaluated by immobilizing the Rh-UTES derivative on porous silicon devices. We found that after immobilization procedure, the Rh-UTES derivate maintained its fluorescent properties. PSi/Rh-UTES' sensing capabilities for Hg^2+^ detection were studied. It was observed that metal-hybrid sensor coordination produces a 0.25-fold enhancement in the integrated fluorescent emission at 6.95 μM Hg^2+^ ion concentration. By comparing the fluorescence response of Rh-UTES derivative in liquid and solid phases, we found that the immobilization procedure produced a 277-fold integrated fluorescence increasing which highlights the benefits of using PSi optical devices as support of the organic receptor. This work may open the door to the development of optical fluorescence-based sensors that can be easily used in field without the need of complicated instrumentation, allowing the fast diagnosis of the quality of natural water sources or water from the industrial waste.

## Abbreviations

ACN: acetonitrile; APTES: 3-aminopropyltriethoxysilane; ATR: attenuated total reflectance; au: arbitrary unities; AuNPs: gold nanoparticles; C: carbon; CDCl_3_: deuterated chloroform; d: double; DMSO: dimethyl sulfoxide; FTIR: Fourier transform infrared; H: high current density; H: proton; IR: infrared spectroscopy; L: low current density; m: multiplet; NMR: nuclear magnetic resonance; PL: photoluminescence; ppm: parts per million; Psi: porous silicon; PSiMc: porous silicon microcavity; q: quartet; Rh-UTES: rhodamine organosilane derivative (3); SD: standard deviation; SEM: scanning electron microscopy; s: singlet; t: triplet.

## Competing interests

The authors declare no competing interests.

## Authors' contributions

GP designed the project, coordinated, reviewed and drafted the manuscript. MDC carried out the main experimental work, and performed the characterizations of interferometry, Infrared, fluorescent spectroscopy, fluorescent microscopy and SEM, and wrote the in liquid phase discussion of fluorescence spectroscopy. AA carried out the organic synthesis, NMR experiments, FTIR and NMR discussion, organized and drafted the manuscript. LHA participated in the PL characterization and results discussion, analysis data, and in drafting the manuscript. ABF performed the fluorescence microscopy analysis and made the tridimensional emission profile through computing data processing. FJMR participated in infrared measurements. All the authors read and approved the manuscript.
